# Extending the Use of Mendelian Randomisation With Non‐Inherited Variants to Assess Socially Transmitted Parental Exposures Under Assortative Mating

**DOI:** 10.1002/gepi.70031

**Published:** 2026-01-21

**Authors:** Benjamin Woolf, Amy Mason, Chin Yang Shapland, Hyunseung Kang, Hannah M. Sallis, Stephen Burgess, Marcus R. Munafò

**Affiliations:** ^1^ School of Psychological Science University of Bristol Bristol UK; ^2^ MRC Integrative Epidemiology Unit University of Bristol Bristol UK; ^3^ MRC Biostatistics Unit University of Cambridge Cambridge UK; ^4^ Department of Public Health and Primary Care, British Heart Foundation Cardiovascular Epidemiology Unit, Victor Phillip Dahdaleh Heart and Lung Research Institute University of Cambridge Cambridge UK; ^5^ Department of Statistics University of Wisconsin‐Madison Madison Wisconsin USA; ^6^ Centre for Academic Mental Health, Population Health Sciences, Bristol Medical School University of Bristol Bristol UK

**Keywords:** Avon Longitudinal Study of Parents and Children (ALSPAC), developmental environment, dynastic effects, gene‐environment correlation, Mendelian randomisation, smoking initiation, social/indirect genetic effects

## Abstract

A longstanding aim of developmental psychology and epidemiology is to understand the causal effects of parental phenotypes on offspring outcomes. Traditional approaches often fail to account for confounding and reverse causation. We evaluate the use of Mendelian randomisation with non‐inherited variants (MR‐NIV) to address these limitations. MR‐NIV leverages non‐inherited genetic variants to instrument the parental phenotype independent of the offspring's genotype. We used Directed Acyclic Graphs and simulations to validate MR‐NIV and explore robustness to assortative mating. In contrast to an alternative MR method which adjusts the parental genotype for offspring genotype, MR‐NIV can be robust to assortative mating when used without trio data. In settings without trio data, MR‐NIV outperformed the adjustment method. The adjustment method outperformed MR‐NIV in settings with trio data. Applying MR‐NIV to the Avon Longitudinal Study of Parents and Children, we assessed the causal effect of parental smoking on offspring smoking initiation at age 16. Results were consistent with observational studies, suggesting a meaningful increase in the risk of offspring smoking due to parental smoking. However, larger sample sizes will be necessary to provide a precise answer. MR‐NIV offers a promising extension of Mendelian randomisation for studying the developmental environment.

## 1 Background

A primary aim of developmental psychology is to understand how childhood experiences influence adult outcomes (Slater and Bremner 2011). Conventional epidemiological approaches to studying developmental influences often fail to account for genetic or environmental confounding (Thapar and Rutter 2019; Rutter 2003; Plomin 2019; Slater and Quinn 2020; Rutter 2007). Parents and offspring share 50% of their genetic variants, as well as numerous environmental exposures. Thus, simple correlational designs can overestimate the causal effect of the parental phenotype on their offspring's phenotype due to genetic confounding and unobserved environmental effects (Harris 2009). For example, the ‘refrigerator mother’ theory of Autism Spectrum Disorder (ASD) posits that a lack of maternal warmth cause an increased risk of ASD in the offspring (Kanner 1968). However, twin studies and other genetically sensitive designs have suggested that this observation may be attributable to genetic confounding (Amaral 2017).

It is generally unethical and/or infeasible to randomise offspring to different developmental environments. Genetically sensitive designs have been used to address this. For example, monozygotic twins raised together have identical germline genotypes and similar childhood environments. A matched comparison of the differences between monozygotic twins cannot be explained by genetic confounding or the shared environment (Barry et al. 2022; McAdams et al. 2021) and thus serves as a valid indication of the influence of development environment. However, monozygotic twin difference designs can fail to provide a representative picture of causal effects in the general population. Furthermore, they do not control for environmental confounders that differ between twins (Keyes and Susser 2022). Sensitivity analyses can be conducted to explore the robustness of results to environmental and genetic confounding but they cannot provide unbiased causal estimates (VanderWeele and Ding 2017).

Mendelian randomisation (MR) is an epidemiological approach that leverages genetic data to derive robust causal effect estimates. Mendel's laws of inheritance state that genetic variants are randomly segregated and independently assorted during meiosis. Genetic variants are thus distributed independently with respect to potential confounders, rendering the random allocation of genetic variants at conception analogous to random treatment allocation in a randomised controlled trial (RCT) (Cornish et al. 2019; Davies et al. 2018). Statistically, MR studies are conducted within an instrumental variables (IV) framework, with genetic variants treated as instruments. An MR analysis provides a valid estimate of the causal effect of the exposure on the outcome if the genetic variant associates robustly with the exposure of interest (the relevance assumption), is independent of any variant‐outcome confounders (the independence assumption) and causes the outcome only via the exposure of interest (the exclusion restriction assumption) (Angrist and Pischke 2009).

In recent years, there has been increasing interest in ‘social genetic effects’ (also called indirect genetic effects, dynastic effects, passive gene‐environment correlation, extended phenotype, and genetic nurture) (Jaffee and Price 2007; Dawkins 2016; Kong et al. 2018). Here, an individual's genotype influences their phenotype, and through their phenotype an individual's genotype thereby influences their environment (Woolf, Sallis, et al. 2022). Specifically, since humans are social animals, the phenotypic expression of an individual's genotype can influence the phenotype of individuals around them. This has empirically been shown to occur using twin studies (Kong et al. 2018). It is thus now recognised that, just as genetic correlations with the environment can inflate environmental estimates, environmental correlations with genetic factors such as social genetic effects are a source of bias in genome wide association studies (GWAS) and post‐GWAS study designs (Howe et al. 2021; Davies et al. 2019).

**Figure 1:**
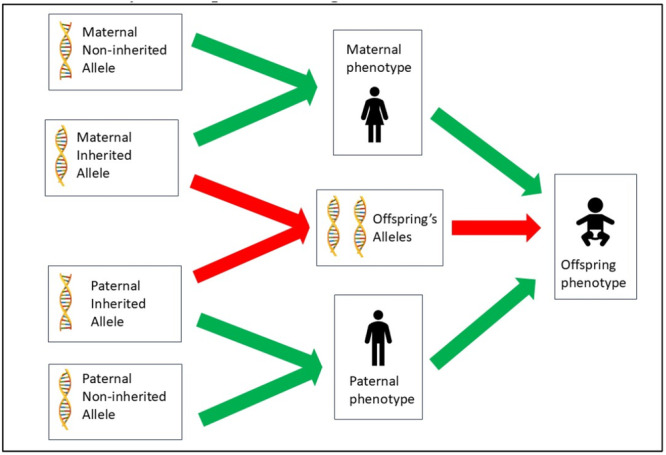
Directed acyclic graph illustrating randomisation to non‐inherited alleles. Parental alleles exert an indirect effect on the offspring via the parental phenotypes, shown by green arrows. Inherited alleles also have a direct genetic effect on the offspring, shown by red arrows. Non‐inherited alleles can therefore be used to specifically assess the effect of the parental phenotype on the offspring phenotype independent of any direct genetic effect.

MR studies typically include only inherited variants (Woolf et al. 2021). However, given that genetic variants are inherited at random, it follows that people are also randomised to the non‐inheritance of variants (Figure 1). These non‐inherited variants can be used to isolate the indirect or ‘social’ genetic effects of parents on their offspring's phenotype, distinct from the direct effects of inherited genetic variants (Zhang et al. 2015). The random non‐inheritance of genetic variants confers a robustness to unmeasured confounding, and the non‐modifiability of genetic variants by the environment confers a robustness to reverse causation (Wootton et al. 2022). Thus, by leveraging parental variants that are not inherited by the offspring, one can use MR with non‐inherited variants (MR‐NIV) to estimate the causal effect of the parental phenotype on their offspring.

One of the earliest applications of non‐inherited (sometimes called non‐transmitted) variants to MR was by Zhang and colleagues who applied them to instrument maternal effects on the offspring during pregnancy (Zhang et al. 2015). Subsequent MR‐NIV studies have also focused on pregnancy related settings (Lawlor et al. 2017; Bond et al. 2022; Richmond et al. 2017; Chen et al. 2020; Warrington et al. 2019; Diemer et al. 2021). The contribution of our paper is to extend the MR‐NIV framework to examine (post‐birth) socially transmitted exposures. These are more complex to study robustly within an MR framework than pregnancy related phenotypes (Hwang and Evans 2020). A specific issue that is assortative mating (when parents select partners who are similar to them) is more likely for socio‐behavioural phenotypes. We therefore examine the use of multivariable analyses with non‐inherited variants to address assortative mating, and introduce the first MR estimator which can address assortative mating with data from only one parent. An alternative approach to MR‐NIV uses both inherited and non‐inherited variants (Havdahl et al. 2023), but adjusts for the offspring genetic risk. We also compare these methods, and clarify when MR‐NIV or this alternative approach should be used.

We first use DAGs to illustrate and describe MR‐NIV. We then use simulations to assess the validity of using the MR‐NIV approach. We compare MR‐NIV to an existing MR approach which instruments the parental phenotype using both inherited and non‐inherited variants but adjusts for the offspring's genetic risk. Finally, we implement MR‐NIV with an applied example in Avon Longitudinal Study of Parents and Children (ALSPAC) by investigating the effect of parental lifetime smoking on offspring smoking status at age 16.

### 1.1 Theoretical Motivation for Mendelian Randomisation With Non‐Inherited Variants (MR‐NIV)

**Figure 2:**
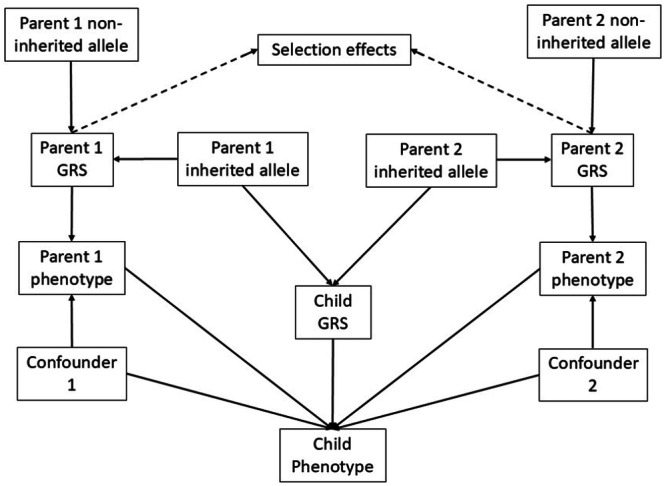
Directed acyclic graph (DAG) of the association between parental phenotype and genotype with the offspring phenotype and genotype. GRS, genetic risk score.

The DAG in Figure 2 can be used to explore possible biases in estimating the causal effect of parents on their offspring, thereby motivating different MR approaches for estimating the parental effects which are summarised in Table 1. If we initially ignore the ‘Selection effects’ variable represented with dashed lines (so that it resembles Figure 1) in Figure 2, then we can see that the non‐inherited parental alleles are valid instruments for the effect of the parental phenotypes on the offspring phenotype. Given the arrow from the non‐inherited alleles, via the parental genetic risk, to the parental phenotype, there should be an association between the two, thereby supporting the relevance assumption. Similarly, because the non‐inherited allele only effects the offspring's phenotype via the parental phenotypes, the exclusion restriction assumption should not be violated by biological pathways in the offspring. Since there are no arrows going into the non‐inherited alleles there are no instrument‐outcome confounders, supporting the independence assumption. Adjusting for the offspring's genetic risk score (GRS) should improve precision in MR‐NIV models without creating risk of bias: the non‐inherited variants are independent of the offspring's GRS but the offspring's GRS influences the offspring phenotype.

**Table 1:** Description of the Mendelian randomisation (MR) estimators with non‐inherited variants (NIV).

Names	Instrument	Exposure(s)	Covariates	Notes
UVMR‐NIV	GRS created from parental non‐inherited variants.	Parental phenotype.	Optional: Child's genetic risk score.	Adjusting for the offspring genetic risk score should improve precision. This method can be used with duo data, but performed less well than multivariable approaches when trio data is available.
UVMR adjusted for child GRS	GRS created from all parental (inherited and non‐inherited) variants.	Parental phenotype.	Non‐optional: Offspring's genetic risk score.	This model is biased if the other parent's phenotype effects the offspring's phenotype (collider bias).
MVMR‐NIV	GRSs created respectively from each parent's non‐inherited variants.	Both parental phenotypes.	Optional: Offspring genetic risk score.	MVMR addresses assortative mating (shown as selection effects in Figure 2) when both parents have been genotyped. However, MVMR‐NIV performed less well than MVMR adjusted for offspring GRS in our simulation in settings with trio data.
MVMR adjusted for offspring GRS	GRSs created respectively from each parent's inherited and non‐inherited variants.	Both parental phenotypes.	Non‐optional: Offspring's genetic risk score.	This method can be used when both parents have been genotyped. By including both parents in a single model, MVMR accounts for assortative mating.
Combining the parental phenotypes	The same as MVMR‐NIV or MVMR adjusted for offspring GRS respectively.	Single variable created as a linear combination of both parental phenotypes.	The same as MVMR‐NIV or MVMR adjusted for offspring GRS respectively.	This method can be used when both parents have been genotyped and parental effects are expected to be homogeneous. It should improve precision compared to modelling each parent separately in an MVMR model when parental sex is not an effect modifier of the effect of the parental‐phenotype on the offspring‐phenotype.
Proxy‐MR‐NIV	One GRS created from non‐inherited variants for the genotyped parent, and one GRS created from inherited variants for the parent who has not been genotyped.^a^	Both parental phenotypes.	Non‐optional: Offspring's genetic risk score.	Proxy‐MR‐NIV can be used (with weak‐instrument robust estimators) when assortative mating is likely and only one parent is genotyped, but requires large samples to produce precise estimates. When a parent has not been genotyped, the variants that the offspring inherited from that parent can be used to proxy that parent's genotype. Proxying their genotype allows estimation of the effect of the genotyped parent independent of the non‐genotyped parent. But, the non‐genotyped parent's estimates cannot be interpreted.

Abbreviations: GRS, genetic risk score; MVMR, multivariable MR; UVMR, univariable MR.

^a^ See Supplementary Methods for a description of how to estimate these from duo data.

In this setting, a traditional observational analysis would be biased. Here, any measurement error in phenotypic confounders (not represented in Figure 2) means there would be both residual confounding and unmeasured confounding via the parental genotypes. Provided the same variants are used in all genetic risk scores, MR‐NIV should be robust to not including all causal variants because missing variants would be independent of non‐included instruments. On the other hand, an equivalent observational analysis which fails to adjust for these variants will suffer from residual genetic confounding.

Assortative mating occurs when individuals mate with partners whose heritable phenotype correlates with their own. Assortative mating can bias MR‐NIV models that include genetic data from only one parent because it induces a correlation between the two parental genotypes, and therefore a backdoor pathway from one parent's genotype to the offspring's phenotype via the second parent's genotype and phenotype. Cross‐trait assortative mating occurs when one partner having higher levels of one trait correlates with the other partner having higher levels of a different trait, such as one partner's BMI being negatively correlated with the other partner's years in education (Border et al. 2022). For simplicity, we show selection effects such as cross‐trait assortative mating occurring on the parental genotypes in Figure 2 rather than on parental phenotype (including related non‐smoking phenotypes in the case of cross‐trait assortative mating).

Multivariable MR (MVMR) is an extension of MR in which two exposures are included within a single IV model (Burgess and Thompson 2015; Sanderson et al. 2019). When a potential biasing pathway is known and can be instrumented, MVMR can be used to attenuate bias (Woolf, Karhunen, et al. 2022; Woolf, Gill, et al. 2022). This comes at the cost of modified identification assumptions. As seen in Figure 2, the back door via the selection effects is blocked by modelling both parents' non inherited variants as instruments for both parents' phenotypes and including the offspring's genotype as a covariate. It is necessary to adjust for the offspring's genetic risk to block the path via the inherited variants to the outcome. Thus, in settings where assortative mating is possible, it is preferable to use an MVMR model which incorporates the effects of both parents' genotypes on the offspring exposure. We will refer to models which include genetic information on both parents as multivariable MR‐NIV (MVMR‐NIV) models. Similarly, we will refer to MR‐NIV models which include genetic information from only one parent as univariable MR‐NIV (UVMR‐NIV) models. Because MVMR estimates direct effects (Carter et al. 2021), MVMR‐NIV will address interference (not illustrated in Figure 2) due to maternal smoking influencing paternal smoking and vice versa. The use of MVMR may result in less precision than an equivalent univariable model. When parental sex is not an effect modifier and genetic risk scores have additive effects, one way of improving precision would be to use a single composite exposure by adding together the level of both parental exposures.

Although MVMR‐NIV can provide a solution to addressing assortative mating, it requires trio data. Several studies have only genotyped one parent and the offspring. Phenotypic trio data may still be available in the absence of trio genetic data because it can be measured reasonably efficiently (Woolf, Pedder, et al. 2022), for example by collecting data on the non‐genotyped parent via genotyped parent‐ or offspring‐based questionnaire. Proxy gene‐by‐environment MR exploits the fact that offspring have inherited variants from their parents to instrument the parental phenotype using the offspring's genotype, in settings where the second parent's genotype is unavailable (Yang et al. 2020). Variants not inherited from one parent and which are not a novel mutation must have been inherited from the other parent, and can therefore be used as proxy of the missing parent's GRS to instrument that parent's phenotype. Adjusting for the offspring's GRS is still necessary to prevent exclusion restriction violations from including the inherited variants. This has the problematic effect of inducing weaker instruments than in any previous MR‐NIV model. In addition, the estimated effect of the non‐genotyped parent on the offspring cannot be interpreted because use of inherited variants means it will be biased by an exclusion restriction violation. As it is an extension of proxy gene‐by‐environment MR, we refer to this approach as ‘Proxy‐MVMR‐NIV’. Proxy‐MVMR‐NIV therefore uses an MVMR model adjusted for both parents' phenotype and the offspring's GRS whilst using inherited variants to instrument the non‐genotyped parental phenotype.

An alternative approach uses both inherited and non‐inherited variants as an instrument in a conventional MR analysis and adjusts for the offspring's genetic risk score (Havdahl et al. 2023). This approach is often easier to implement than MR‐NIV because the default option in many packages is to create the genetic risk scores for the parent and offspring with both inherited and non‐inherited variants (sean‐harrison‐bristol 2022; Purcell et al. 2007; Choi and O'Reilly 2019). However, as seen in Figure 2, the offspring's genetic risk is influenced by both parents' genetic risk and so naïve adjustment for the offspring's genetic risk in a univariable MR model introduces collider bias between both parents' genetic risks, even in the absence of assortative mating. Thus, in contrast to MR‐NIV, univariable implementation of this method (e.g., in settings without trio data) is biased by the effect of the second parent on the offspring's phenotype. Non‐MR analyses using inherited and non‐inherited variants in GRSs have been found to be better powered than those only using non‐inherited variants (Tubbs et al. 2020)

Pleiotropy, when a variant impacts on multiple phenotypes independently, is a threat to the validity of all MR studies. Standard sensitivity analyses have been developed, and should be used, to detect pleiotropy (Sanderson et al. 2022). Pleiotropy ‘robust’ estimators can also be used to relax the exclusion restriction assumption (Slob and Burgess 2020). However, studying (post pregnancy) socially transmitted effects using non‐inherited alleles should offer some protection to pleiotropy because the exclusion restriction violations can generally only occur through socially mediated pathways. Genetic variants are also only random conditional on the genotype from which they were inherited from (Davies et al. 2019). As such, the non‐inherited variants will generally be only approximately random. Standard approaches for addressing population structure (such as restricting to a relatively homogeneous population and adjusting for principal components of ancestry) should thus still be employed when applying MR‐NIV.

### 1.2 Simulation Study to Explore the Performance of MR‐NIV

#### 1.2.1 Overview of Simulation

We report our simulations using the ADEMP (aims, data‐generating mechanisms, estimand, methods, and performance measures) approach (Morris et al. 2019). Further details about the simulation's data‐generating mechanism and methods can be found in the Supporting Methods Section 1.

##### 1.2.1.1 Aims

To assess the performance of various Mendelian randomisation methods in estimating effects of parents on their offspring.

##### 1.2.1.2 Overview of the Data‐Generating Mechanisms

We ran a simulation of the DAG in Figure 2 in a sample of approximately 10,000 trios, as a simulated dataset with similar characteristics to the ALSPAC dataset with no missing data or loss to follow‐up. Our simulation considered four scenarios in which we varied the presence of a (homogeneous) parental causal effect and assortative mating between the parents:

- A causal effect and no assortative mating.
- A causal effect and assortative mating.
- No causal effect and no assortative mating.
- No causal effect and assortative mating.

The parameters in the simulation were chosen so that the GRS explained 0.5% of the variability, a similar proportion of the phenotypic variance as the genome‐wide significant variants for lifetime smoking index. We simulated the causal effect (when present) to be reasonably large (standardised mean difference = 0.75); but (as is arguably common for many psychological and behavioural phenotypes; Harris 1995) the vast majority (> 90%) of variation in the offspring's phenotype is explained by non‐genetic and non‐parental factors such as environmental confounding.

##### 1.2.1.3 Estimand

The estimand was the average causal effect of the parental exposure phenotype on the offspring's outcome phenotype.

##### 1.2.1.4 Overview of the Methods

In each scenario we compare four approaches implemented using two‐stage least squares regression: UVMR‐NIV, MVMR‐NIV, UVMR, and MVMR with GRSs created with inherited and non‐inherited variants adjusted for offspring GRS (see Table 1).

However, MR‐NIV is likely limited in practice by a lack of trio data and/or limited power. We therefore:

A. Explored (using the same data‐generative model) if power when there is a casual effect can be improved by combining the parental phenotypes into a composite variable when trio data is available.
B. Examine the use of Proxy‐MVMR‐NIV to address assortative mating when trio data is not available in the settings with assortative mating.

Owing to expected weak‐instrument bias, we implement Proxy‐MVMR‐NIV using four weak‐instrument robust estimators using summary data described in the Supporting Methods Section 3 (Continuously updating Generalised Methods of Moments [GMM], debiased IVW, GRAPPLE [a multivariable extension of the MR‐RAPS method], and QHet) instead of 2SLS regression. We additionally explore how Proxy‐MVMR‐NIV performs in a simulated dataset of similar size to the Norwegian Mother, Father, and Child Cohort Study (MoBa) dataset (*n* ~ 100,000 duos).

##### 1.2.1.5 Performance Measures

The methods are compared on their mean bias, 95% coverage of the estimates (i.e. the proportion of datasets for which the 95% CI includes the true effect, which is a measure of how accurate the CI are; Morris et al. 2019), variance of the effect estimates across datasets, and mean 95% confidence interval width for their estimate of the average causal effect of parental phenotype on the offspring's phenotype over 1000 iterations of the simulation. Due to the computational time required to estimate the uncertainty for the estimators (over an hour per iteration for QHet on the University of Bristol super computer), we only explore how the QHet estimator implementation of Proxy‐MVMR‐NIV performs in terms of bias.

#### 1.2.2 Results of the Simulation Study

##### 1.2.2.1 Causal Effect and No Assortative Mating

Table 2 and Supporting Table S1 present the results of the simulations where the parental phenotype does cause the offspring's phenotype. In this setting, univariable MR‐NIV is unbiased, and has good coverage. As expected, univariable MR using parental inherited and non‐inherited variants adjusted for the offspring's GRS is biased and had low coverage – likely due to the offspring's GRS being a collider of the association between the parental‐phenotype and offspring‐phenotype.

**Table 2:** Results of simulation when the parental phenotype does cause the offspring's phenotype.

Assortative mating	Method	Bias	Variance of estimates	Coverage	CI width
No assortative mating	UVMR‐NIV	0.002	0.015	0.950	0.463
	UVMR adjusted for offspring GRS	−0.239	0.010	0.328	0.401
	MVMR‐NIV	−0.005	0.007	0.953	0.326
	MVMR adjusted for offspring GRS	−0.004	0.005	0.952	0.280
Assortative mating	UVMR‐NIV	0.502	0.027	0.140	0.628
	UVMR adjusted for offspring GRS	0.330	0.017	0.301	0.514
	MVMR‐NIV	−0.002	0.040	0.973	0.798
	MVMR adjusted for offspring GRS	−0.002	0.012	0.952	0.435

*Note:* For simplicity of presentation, results here are the average of both parents. UVMR and MVMR adjusted for offspring GRS included both inherited and non‐inherited genetic variants. Results stratified by parent can be found in Supplementary Table S1.

MVMR‐NIV performed similarly to UVMR‐NIV. No bias was observed and coverage was high for the approach using an MVMR model with both parents' inherited and non‐inherited variants adjusted for the offspring's GRS. This is consistent with collider bias mediated by the other parent's phenotype with UVMR, producing an inferior performance with this approach. In addition, both multivariable approaches had more precise estimates than their univariable equivalent. This likely represents greater variation in the offspring's phenotype being explained by including the other parent. MVMR‐NIV was the less precise multivariable approach.

##### 1.2.2.2 Causal Effect and Assortative Mating

As expected, both univariable approaches were biased and had low coverage in the presence of assortative mating. The multivariable approaches, on the other hand, had low bias and improved coverage. However, the MVMR‐NIV models tended to have substantially wider 95% CIs than the univariable MR‐NIV models in this setting. MVMR using the parental inherited and non‐inherited variants adjusted for the offspring's GRS was similarly low bias to MVMR‐NIV but had tighter CIs than both its univariable implementation and both MR‐NIV models.

##### 1.2.2.3 No Causal Effect and No Assortative Mating

The results of the setting with no causal effect can be found in Supporting Table S2. For the two multivariable approaches, the results in the setting with no causal effect are almost identical to the results in the previous setting with an effect and no assortative mating. However, since the offspring's genotype is no longer a collider between the parental genotype and the offspring's phenotype, the UVMR model including the parental inherited and non‐inherited variants adjusted for the offspring's GRS is not biased in this setting.

##### 1.2.2.4 No Causal Effect and Assortative Mating

The results for the multivariable approaches were similar to the setting with assortative mating and a causal effect. However, under the null, the univariable approaches were no longer biased by assortative mating and maintained good levels of coverage. This is because unlike in the setting with a causal effect, there is no backdoor path from the other parent's phenotype to the offspring's phenotype. In addition, the univariable approaches had tighter 95% CIs than the multivariable approaches, with UVMR‐NIV being the less precise of the two univariable approaches.

##### 1.2.2.5 Improving Precision by Combining the Parental Phenotypes

The 95% CI width of the MVMR‐NIV estimates can be reduced by using the sum of both parental phenotypes as the exposure variable (Supporting Table S3). For example, in the presence of assortative mating and a causal effect, the MVMR‐NIV model with the summed exposure had an average 95% CI width less than half that of the equivalent MVMR‐NIV model which includes each parental phenotype as a separate exposure. As expected, these models had high coverage and low bias.

##### 1.2.2.6 Using Proxy‐MVMR‐NIV to Address Assortative Mating in Settings Without Trio Data

Often genetic data is not available for trios, but assortative mating is plausible. Here, one can theoretically use variants that were not inherited from the genotyped parent to proxy for the genotype of the non‐genotyped parent, in what we describe as a ‘Proxy‐MVMR‐NIV’ analysis.

Despite implementing Proxy‐MVMR‐NIV with weak‐instrument robust methods (QHet GMM, debiased IVW, and GRAPPLE), Proxy‐MVMR‐NIV appeared to suffer from substantial weak‐instrument bias in the simulation with an ALSPAC sized (10,000 families) sample (Supporting Table S4). However, weak‐instrument bias was ameliorated (Table 3 and Supporting Table S5) in an additional simulation which increased the sample size 10‐fold to be in line with the MoBa dataset (~100,000 duos). Indeed, with a MoBa‐sized sample, bias in QHet and GMM was around 20‐fold smaller than a naive MR‐NIV model. Overall, GMM appeared to be the best estimator in terms of minimising bias and increasing precision.

**Table 3:** Results of the simulation using Proxy‐MVMR‐NIV when there is assortative mating in a MoBa (~100,000 duos) sized sample.

Method	QHet		GMM		Debiased IVW		GRAPPLE	
Causal effect is	Non‐null	Null	Non‐null	Null	Non‐null	Null	Non‐null	Null
Bias	0.074	0.065	0.047	0.023	0.394	0.473	−0.064	0.205
Coverage	NA	NA	1.000	0.876	0.789	0.987	0.971	0.971
95% CI width	NA	NA	0.410	0.215	110,873.5	119,433.6	165.794	114.723
Variance of estimates	0.007	0.007	0.002	0.005	4372.346	5899.798	4.595	3.094

*Note:* For simplicity of presentation, results here are the average of both parents. Results stratified by parent can be found in Supplementary Table S5.

### 1.3 Applied Example in ALSPAC

#### 1.3.1 Background to the Applied Example

Smoking is a leading cause of preventable deaths (He et al. 2022) and global sales of cigarettes continue to grow (Statista Internet 2023). Smoking is highly addictive (Stolerman and Jarvis 1995), and many people start smoking at a young age (Forey et al. 2002). Therefore, preventing or delaying smoking initiation could substantially reduce smoking related morbidity and mortality. Indeed, the majority of smokers have, historically, started to smoke in adolescence (Giovino et al. 1995). Many studies have sought to establish the causes of smoking in adolescence with the hope of informing health policy (Conrad et al. 1992; McCaul et al. 1982). A consistent and reproducible finding in the literature is that parental smoking correlates with offspring smoking (Rantakallio 1983; Croft et al. 1985; Borland and Rudolph 1975; Bailey et al. 1993; Loke and Wong 2010; Harakeh et al. 2010; Sargent and Dalton 2001; Scragg et al. 2003; Otten et al. 2007; Jackson and Henriksen 1997; Bauman et al. 1990). For example, a recent meta‐analysis found that having one smoking parent was associated with a 1.7 fold increase in the odds of an offspring smoking, and having both parents smoke was associated with a 2.7 fold increase (Leonardi‐Bee et al. 2011).

One hypothesis is that parental smoking causes offspring smoking through offspring observing parental smoking behaviour and then modelling their own smoking behaviour on parental behaviour (Vitória et al. 2020; Alves et al. 2017). Since many studies are prospective (Conrad et al. 1992; Chassin et al. 1986; Biglan et al. 1995; Doherty and Allen 1994; Bricker et al. 2003; Engels et al. 1999; Bricker et al. 2006; Peterson et al. 2006; Mays et al. 2014), it is unlikely that the association between parental and offspring smoking is attributable to reverse causation or selection bias. However, these studies are susceptible to confounding, for example from the shared environment or genetic confounding. We therefore apply MR‐NIV to assess the existence of a causal effect of parental lifetime smoking on early onset smoking of their offspring. Early onset smoking was defined by their smoking status at age 16 and estimated in the Avon Longitudinal Study of Parents and Children (ALSPAC).

#### 1.3.2 Applied Example Methods

We illustrate the use of MR‐NIV by investigating the causal effect of parental smoking on offspring smoking in ALSPAC. We report this analysis using the STROBE‐MR guidelines (Skrivankova et al. 2021), although further details for some sections can be found in the Supporting Methods Section 2.

##### 1.3.2.1 Summary of the Alspac Sample

ALSPAC is a birth cohort study on around 14,000 individuals from Avon (UK) with expected due dates between April 1991 and December 1992 and their parents (Boyd et al. 2013; Fraser et al. 2013; Northstone et al. 2023). The ALSPAC study website contains details of all the data that is available through a fully searchable data dictionary and variable search tool (Bristol U of Explore data and samples Internet 2025). We restricted the ALSPAC sample to self‐reported Europeans, and excluded partners who the mother did not report to be the offspring's father. The Supporting Methods Sections 2.1 and 2.2 provide further details on ALSPAC and the specific measurements used here.

##### 1.3.2.2 Instrument Construction

Smoking is a multidimensional trait, with dimensions including smoking initiation, duration, heaviness, and cessation. The transmissibility of smoking can be potentially dependent on (having measured) a specific dimension (Mays et al. 2014; Gilman et al. 2009). To instrument smoking we therefore use the 126 independent (clumping *r* ^2^ = 0.001 and a distance of 10 Mb) variants which are genome‐wide significantly associated with lifetime smoking index in the UK Biobank (Wootton et al. 2020), and used the same GWAS to extract the regression‐beta weights used to weigh the variants in the weighted GRS. Lifetime smoking index is a broad measure of smoking behaviour which aims to capture variability in smoking behaviour due to its different dimensions.

The weighted GRSs were manually created independently for each parent and the offspring using the algorithm described in Supporting Table S6. We included variants which have high certainty of not being inherited in the GRS used to instrument parental smoking, measured when the offspring was aged 12. An example of a high confidence non‐inherited allele would be one where the parent of interest has at least one risk allele but the offspring has none. Since the offspring does not have the risk allele, they cannot have inherited that one (or more) that the parent has. Variants for which it is uncertain which parent they were inherited from (e.g., when both parents and the offspring have one effect allele) are not included in the GRS for that set of individuals.

##### 1.3.2.3 Overview of the Analysis Plan

We assess if genetic predictors of parental smoking behaviour when the offspring was aged 12 associated with the odds of the offspring having started smoking by age 16. Our primary MR analysis was implemented using a Wald ratio of the regression of the offspring's smoking at aged 16 on the parental non‐inherited GRS divided by the regression estimate of parental smoking when the offspring is aged 12 on the parental non‐inherited GRS. The Wald ratio is equivalent to 2SLS regression with a single instrument, but facilitates implementing a logistic link function. For offspring who only had data on one parent, we implemented the analysis using genetic and phenotypic data from only mother's or father's respectively. These analyses correspond to the univariable MR‐NIV analyses in the simulation study and Table 1. When genetic data was available on both parents, we combined the parental phenotypes into a single variable, and summed both individual parental GRSs into a single combined parental GRS. By doing so, this analysis corresponds to the approach which combines the parental phenotypes discussed in the simulation study and Table 1. We also assess the phenotypic association between parental and offspring smoking using conventional logistic regression.

##### 1.3.2.4 Positive Control Outcome

Unlike a negative control, the role of our positive control outcome is not to assess the validity of the IV assumptions but instead to calibrate power in ALSPAC (Woolf and Burgess 2025). We use parental hypertension at the same time point as when parental smoking was measured (offspring aged 12) as a positive control outcome because there is strong evidence from a range of sources that smoking has a causal effect on blood pressure (Woolf et al. 2023; Xia et al. 2022; Narkiewicz et al. 2005).

##### 1.3.2.5 Missing Data and Multiple Imputation

Due to high expected phenotypic missingness, we perform the analyses both with and without multiple imputation. We did not impute genetic data for non‐genotyped individuals (e.g. using their relatives), who may not have consented to the use of their genetic data. The analysis was therefore structured into families who had trio genetic data, families who had maternal but not paternal genetic data, and families who had paternal but not maternal genetic data. We meta‐analyse these three estimates to derive an overall test of the impact of parental smoking.

##### 1.3.2.6 Sensitivity Analyses

To address weak‐instrument bias, we use three weak‐instrument robust estimators (MR RAPS, GMM, and debiased IVW described in the Supporting Methods Section 3) in sensitivity analyses. These are the univariable MR analogues of the multivariable weak instrument robust estimators we applied to Proxy‐MVMR‐NIV in the simulation study. We assess weak‐instrument bias using first stage F‐statistics and also explore the heterogeneity in variant specific MR estimates to assess pleiotropy.

#### 1.3.3 Results of the Applied Analysis

We analysed data on 6962 unrelated individuals in the offspring generation for whom we had genetic information on both the individual and at least one parent. Of these 5345 had genetic information on mothers but not fathers, 494 on fathers but not mothers, and 1123 on both mothers and fathers. Demographic information on each sub‐sample can be found in Supporting Table S7. This table shows that almost all covariates (e.g., sex, parental socio‐economic position or education) have a moderately sized association (e.g., OR < 0.95 or OR > 1.05) with offspring smoking except for parental age. Although there was evidence of a strong correlation between maternal and paternal smoking (*r* = 0.33, 95% CI: 0.30–0.36) among those with genetic information among both parents), there was not evidence supporting a strong genetic correlation between the maternal and paternal genetic liability to lifetime smoking (*r* = 0.03, 95% CI: −0.03 to 0.09) using the genetic risk scores. This suggests that assortative mating is not substantial in this sample.

**Figure 3:**
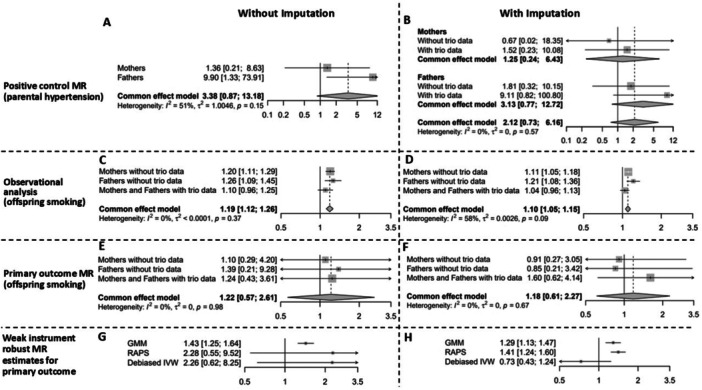
Results of the analysis in ALSPAC. Estimates represent the odds of the offspring smoking at age 16 (or parental hypertension status for positive control analyses) per one additional pack per week smoked by a parent when the child was aged 12. (A) Meta‐analysis of the (positive control analysis) MR of each parent's smoking behaviour on that parent's hypertension status using non‐imputed data. We created three imputed data sets: one for parents with trio genetic data, one for mothers without trio data, and one for fathers without trio data. (B) Meta‐analysis of the (positive control analysis) MR of each parent's smoking behaviour on that parent's hypertension using imputed data. We created three imputed data sets: one for parents with trio genetic data, one for mothers without trio data, and one for fathers without trio data. We derive overall estimates for mothers (and fathers) analogous to the parent specific estimates in 3 A by meta‐analysing the estimate for that parent from the data set with and without trio data. (C, D) Observational analysis of parental smoking behaviour on offspring smoking status aged 16 without and with imputation respectively. (E, F) Meta‐analysis of the (primary analysis) MR of parental smoking behaviour on offspring smoking status aged 16 without and with imputation respectively. To improve precision, the exposure and instrument for the analysis using trio data were the summation of both parents' instruments and exposure statuses respectively (analogous to the approach which combined the parental phenotypes in the simulation study and Table 1). (G, H) Meta‐analysis results of the (primary analysis) using weak‐instrument robust estimators.

Figure 3 presents the results for the complete case analysis and the analysis with imputed missing phenotypic data (exposure, outcome, and—for the non‐genetic observational analysis—covariate). In both the non‐imputed (Figure 3A) and imputed (Figure 3B) meta‐analyses of the positive control MR estimates, using a Wald ratio, we did not identify strong evidence of an association between parental genetically predicted lifetime smoking and parental hypertension when the offspring was 12 years old (OR = 3.38, 95% CI: 0.87–12.18, *n* = 5263 without imputation; and OR = 2.12, 95% CI: 0.73–6.16, *n* = 6962 after imputation). This indicates that our subsequent MR analysis is likely to have imprecise estimates. In these and all subsequent MR analyses, the OR between genetically predicted lifetime smoking index and outcome phenotypes was scaled to represent the odds of the offspring smoking at age 16 per one additional pack smoked per week by a parent when the offspring was aged 12.

The unadjusted non‐genetic analysis (using unimputed data, Supporting Table S7) found that each pack per week smoked by the mothers when the offspring were aged 12 was associated with a 1.12‐fold (95% CI: 1.07–1.18) increase in the odds of the offspring smoking aged 16; the corresponding estimate for fathers was 1.11 (95% CI: 1.07–1.15). Estimates for the combined association in mothers and fathers remained similar after adjustment for covariates (OR = 1.19, 95% CI: 1.12–1.26, *n* = 2108 without imputation, Figure 3C; and OR = 1.10, 95% CI: 1.05–1.15, *n* = 6962 after imputation, Figure 3D).

The MR estimates from the primary analysis (OR = 1.22, 95% CI: 0.57–2.61, *n* = 3396 without imputation, Figure 3E; and OR = 1.18, 95% CI: 0.61–2.27, *n* = 6962 after imputation, Figure 3F) are consistent with the observational estimates. However, instrument strength was comparatively low in these analyses: the *F*‐statistic with non‐imputed data was 4 and 6 when using imputed phenotype data.

Sensitivity analysis using weak‐instrument robust estimators found larger effects than those in the primary (Wald ratio) analysis (Figure 3G,H, Supporting Figure S1) with the exception of debiased IVW with imputed phenotype data. Similar to the simulation, the estimates for GMM are an order of magnitude more precise than the other estimates. Table 3 does not, however, imply that this is at the cost of greater bias. Combined with the similar results from MR RAPS and the high variance of Debiased in simulation (Table 3), this implies that the divergent results for Debiased IVW in Figure 3H should be treated with caution. Finally, we note that there was not convincing evidence of heterogeneity in *I* ^2^ statistics for the SNP‐specific Wald ratios (Supporting Table S8).

## 2 Discussion

### 2.1 Key Results and Interpretation

Residual confounding limits many observational approaches to studying the developmental environment. Offspring inherit a random half of their own parents' genome. However, the half of parental variants which their offspring do not inherit cannot have a direct genetic effect on their offspring's phenotype. An association between parental non‐inherited variants and an offspring's outcome therefore cannot be explained by any biological effect of the variants in the offspring. However, because the inheritance of a particular variant by the offspring was random, it should be independent of environmental confounders (Smith et al. 2007). As such, the most plausible interpretation of an association is that the parental phenotype caused the offspring outcome of interest. In this study we therefore assessed the use of non‐inherited variants as an approach to instrumenting parental phenotypes and therefore to use Mendelian randomisation analyses to study effects of the familial environment. Through the interrogation of DAGs and the use of simulations, we argue that, given large enough sample sizes, MR‐NIV can provide reasonably unbiased estimates of the effect of the parental phenotype on their offspring phenotype.

Smoking is both highly dangerous and socially transmissible; indeed, parental smoking has been proposed as an important risk factor for offspring smoking initiation (Vitória et al. 2020; Alves et al. 2017). We therefore explored the impact that parental smoking has on offspring smoking status when aged 16 using the ALSPAC sample. Our MR‐NIV model found evidence consistent with the findings of the observational model that parental smoking results in a large (20%) increase in the risk of offspring smoking initiation at age 16. Although our primary MR‐NIV estimates were imprecise, the failure to detect a consistently association in the positive control meta‐analysis and the tighter CI when using weak‐instrument robust estimators are compatible with this being due to low power rather than an absence of a true effect.

An alternative method to MR‐NIV uses both inherited and non‐inherited parental variants (including inherited variants) but adjusts for the offspring's GRS. Our simulations showed that, when trio data is available, this alternative approach performed better than MR‐NIV. The advantage of MR‐NIV is that it does not require trio data. To our knowledge, Proxy‐MVMR‐NIV is the only design‐based approach for addressing assortative mating with duo data. Collecting trio data is both harder (e.g., due to requiring informed consent on three people) and more expensive than duo data. Given that MR studies tend to require much larger sample sizes to achieve the same power as an observational study (Burgess 2014), meta‐analyses of multiple data sources may be required to achieve sufficient precision to detect treatment effects. Indeed, the failure of the positive controls suggests that there is insufficient power in the ALSPAC sample (e.g. due to self‐reporting error and/or small samples) to detect relevant effects in our MR analysis.

As expected, our simulations found that (all else being equal) Proxy‐MVMR‐NIV suffered from much more weak‐instrument bias than (MV)MR‐NIV. Weak‐instrument bias can, to some extent, be attenuated by increasing sample sizes. As such, we found that Proxy‐MVMR‐NIV performed better in a MoBa sized sample than an ALSPAC sized sample. However, this does not imply that all biases in Table 2 (and Supporting Tables S1 and S2) can be addressed by increasing sample size. Weak‐instrument bias likely explains why no estimator achieved precisely no bias in any simulation. The larger biases with the univariable approaches shown in Table 2 have structural causes (e.g. collider bias and assortative mating), and therefore will not be attenuated by larger samples alone.

### 2.2 Strengths and Limitations

Mendelian randomisation studies which do not condition on the parental genotype can be subject to some residual confounding from population structure, assortative mating, or dynastic effects (Davies et al. 2019). MR‐NIV studies instrument dynastic effects and so should be less prone to bias from these sources. However, unless grandparental genetic data are included, the design could still be impacted by population structure. We attempted to address population structure by restricting our analysis to participants whose parents' self‐reported as European ancestry and adjusting for parental principal components of ancestry. Smoking is known to be a phenotype for which assortative mating occurs (Agrawal et al. 2006), but fewer than 1500 trios were genotyped. Assuming that families with trio information generalise to the rest of the sample, the low correlation between maternal and paternal genetic risk scores in ALSPAC implies that assortative mating may not be a major source of bias our MR analysis. Similarly, the MVMR‐NIV estimates in the parents with trio data tended to be similar to the UVMR‐NIV estimates in the parents without trio data. Although we could have employed Proxy‐MVMR‐NIV, in light of our simulation results we reasoned that estimates would be unreliable in an ALSPAC sample size.

A second potential limitation is ‘interference’ (Hudgens and Halloran 2008), when another individual's exposure influences the exposure‐outcome association of interest. This will occur, for example, if there is a bidirectional feedback loop due to offspring smoking also influencing parental smoking. This can both complicate attempts to estimate the effects of a specific individual, and introduce exclusion restriction violations. One advantage of studying socially transmittable phenotypes using an MR framework is that the offspring's smoking would need to feedback to the parental non‐inherited variants rather than parental smoking in order to bias the analysis (Burgess et al. 2015). Since the parental genetic variants are fixed at their conception (which is by definition before their offspring can smoke), MR‐NIV analyses should not be biased by such feedback loops.

A third limitation of the applied analysis is that within the ALSPAC sample the mother's partner at the time of completing the questionnaire is not necessarily the same partner from whom genetic data was collected. We do not expect the inclusion of genetic information from non‐biological parents to bias our analysis since all adoptive parent's variants are in theory non‐inherited. However, changes in the mother's partner over time would reduce precision and increase weak‐instrument bias.

In the applied analysis, we were forced to restrict the ALSPAC sample to families with genetic data on at least two individuals. As such, there is risk of selection bias with those consenting to be genotyped likely to be non‐random. Likewise, among included families, there was missing phenotypic data. This can result in a selection bias in the complete case analysis. Although we attempt to mitigate this using multiple imputation, imputation can produce more biased estimates than complete case analyses when the outcome is missing not at random (Hughes et al. 2019).

Variants which associate with smoking traits, such as lifetime smoking index, have been shown to associate with a number of potentially pleiotropic phenotypes, such as alcohol consumption (Reed et al. 2025). This could plausibly introduce bias into our analysis, for example if parental alcohol consumption increases offspring smoking independent of parental smoking. However, we did not observe evidence supporting pleiotropy in our heterogeneity tests as displayed in Supporting Table S8.

Finally, the precise results of our simulation arise from the specific parameters simulated and may not therefore generalise to all applied settings.

### 2.3 Conclusion

In this study we have evaluated the use of Mendelian Randomisation with Non‐Inherited Variants as an approach to assess the causal effects of parental phenotypes on offspring outcomes. Our theoretical exploration using Directed Acyclic Graphs (DAGs) and simulations demonstrated the validity of the MR‐NIV approach. MR‐NIV underperformed the existing approach which adjusted the parental genotype for the offspring genotype in settings with trio data. However, an advantage of MR‐NIV over the adjustment method is that it can address assortative mating in the absence of trio data. The approach requires large sample sizes to achieve sufficient precision, as evidenced by our applied analysis. The applied MR analysis of parental smoking on the risk of offspring smoking initiation at age 16 provides consistent, if imprecise, results with the observational finding supporting an effect of parental smoking on offspring early onset smoking.

## Ethics Statement

Informed consent for the use of data collected via questionnaires and clinics was obtained from participants following the recommendations of the ALSPAC Ethics and Law Committee at the time. Consent for biological samples has been collected in accordance with the Human Tissue Act (2004). Ethical approval for the study was obtained from the ALSPAC Ethics and Law Committee and the Local Research Ethics Committees.

## Conflicts of Interest

The authors declare no conflicts of interest.

## Use of Large Language Models

No large language models were used in the writing of the main text of this manuscript and its appendix, however a first draft of the abstract was written by Microsoft Co‐pilot.

## Supporting information


**Figure S1:** Weak‐instrument robust estimators for the primary analysis in ALSPAC. **Table S1:** Results of simulation when the parent's phenotype does cause the child's phenotype. Standard errors are Monte‐Carlo standard errors. **Table S2:** Results of simulation when the parent's phenotype does not cause the child's phenotype. Standard errors are Monte‐Carlo standard errors. **Table S3:** Results of MVMR‐NIV using a composite parental exposure. **Table S4:** Results of Proxy‐MVMR‐NIV in settings with assortative mating in an ALSPAC scale sample. **Table S5:** Results of Proxy‐MVMR‐NIV in settings with assortative mating in an MoBa scale sample. **Table S6:** Algorithm used to decide if an allele was inherited or not. **Table S7:** Descriptive results from ALSPAC. **Table S8:** Heterogeneity (I^2^ statistics) for the SNP specific Wald ratios for the primary MR analyses.

## Data Availability

The data used in this study is available via application directly to ALSPAC (proposal number B4180). Access is subject to an approved proposal and payment of a data access fee. Proposals can be submitted via the study website (http://www.bristol.ac.uk/alspac/). All analyses were performed using R (R Core Team 2021). Multiple imputation was performed using the MICE R package (Buuren et al. 2022). Estimators were implemented using the AER, MVMR, MendelianRandomization, mr.divw, GRAPPLE, ivmodel, and RAPs packages (Mendelian Randomization 2024; Kleiber and Zeileis 2008; Sanderson et al. 2021; Wang et al. 2021; Wu et al. 2024; Kang et al. 2021; Zhao et al. 2020). Meta analyses were performed using the meta R package (Harrer et al. 2021). Code used in the study is available at 10.17605/OSF.IO/RHU6P.
