# Supplementary material for: Extending the Use of Mendelian Randomisation With Non‐Inherited Variants to Assess Socially Transmitted Parental Exposures Under Assortative Mating
Source: Genet Epidemiol. 2026 Jan 21;50(1):e70031. doi: 10.1002/gepi.70031 (PMC12820921; doi:10.1002/gepi.70031)
Supplement: Supplementary file 1 — Figure S1: Weak‐instrument robust estimators for the primary analysis in ALSPAC. Table S1: Results of simulation when the parent's phenotype does cause the child's phenotype. Standard errors are Monte‐Carlo standard errors. Table S2: Results of simulation when the parent's phenotype does not cause the child's phenotype. Standard errors are Monte‐Carlo standard errors. Table S3: Results of MVMR‐NIV using a composite parental exposure. Table S4: Results of Proxy‐MVMR‐NIV in settings with assortative mating in an ALSPAC scale sample. Table S5: Results of Proxy‐MVMR‐NIV in settings with assortative mating in an MoBa scale sample. Table S6: Algorithm used to decide if an allele was inherited or not. Table S7: Descriptive results from ALSPAC. Table S8: Heterogeneity (I2 statistics) for the SNP specific Wald ratios for the primary MR analyses. [file GEPI-50-0-s001.docx]

**Supplementary Methods**

1. Simulation study

*1.1. Further details on the Data-generating Mechanisms*

The data-generative model is presented by the Directed Acyclic Graph (DAG) in Figure 2. More formally, we simulated 126 single nucleotide polymorphisms (SNPs) for each parent to represent the genetic liability toward the exposure. Each parental SNP was simulated as the sum of two independent and identically distributed binomial variables which represented alleles with the following parameters:

MAF ~ TN(0.58, 0.21^2^)

Ai ~ B(1, MAF)

Ani ~ B(1, MAF)

SNP = Ai + Ani

where MAF represents the minor allele frequency, Ai represents the allele that was inherited by the offspring, and Ani represents the allele that was not inherited by the offspring. TN stands for a truncated normal distribution, with a minimum value of 0 and a maximum value of 1. The distribution of minor allele frequencies, and number of SNPs, was based on the independent genome wide significant SNPs in the Genome Wide Association Study (GWAS) of lifetime smoking by Wootton and colleagues (1).

Each SNP then had a unique effect on the parental phonotype which were simulated as independent and identically distributed normal variables:

BETA ~ N(0.2, 0.03^2^)

The distribution of beta effects was loosely based on the standardised mean differences for the genome wide significant SNPs in the GWAS of lifetime smoking (1). Thus, first parent’s genetic liability to the exposure was then simulated as the sum of their allele score multiplied by the weighting for each allele:

P1GR = $\sum_{i}^{126} {BETA}_{i-P1}*{SNP}_{i-P1}$

P2GR = $\sum_{i}^{126} {BETA}_{i-P2}*{SNP}_{i-P2}$

where ${SNP}_{i-P1}$ represents the first parent’s i^th^ SNP, and ${BETA}_{i-P1}$ represents the BETA parameter for the i^th^ SNP of the first parent, and ${BETA}_{i-P2}$ and ${SNP}_{i-P2}$ represent the same for the second parent. We thus allowed, for any specific variant, the exact effect of the variant on each parent’s phenotype to differ. This is to reflect the argument that variant-smoking effects should differ between sex’s (2). However, because all effects are sampled from the same distribution (BETA), this decision to allow effects for a specific SNP to vary between sex’s should not impact on the results of genetic risk score analyses.

The child’s alleles were also allowed to have different weights to that of their parent’s because variant-smoking effects may differ between generations (2). Again, since child and parental effects are all sampled from the same distribution (BETA) this should not impact on results using genetic risk scores. Thus, the child’s genetic liability to the exposure was simulated as:

CGR = $\sum_{i}^{126} {BETA}_{i-1}*{Ai}_{i-P1}+ {BETA}_{i-2}*{Ai}_{i-P2}$

…where ${Ai}_{i-P1}$and ${Ai}_{i-P2}$ represent the alleles inherited from the first and second parent respectively, and ${BETA}_{i-1}$ and ${BETA}_{i-2}$ represent the allele’s effects.

We simulated two confounders of the parent-offspring environmental effect as two independent and identically distributed normal distributions:

C1 ~ N(0,1^2^)

C2 ~ N(0,1^2^)

The first parental phenotypes were then simulated as:

PP1 = P1GR + C1*6 + ε_1_

PP2 = P2GR + C2*6 + ε_2_

where ε_1_ and ε_2_ are error term simulated as independent and identically distributed normal distributions such that ε ~ N(0, 5^2^). The standard deviation of 5 for the error term was chosen so that the r^2^ of each of the genetic risk score would be around 0.5%. This is an approximation of the r^2^ of lifetime smoking by the independent genome wide significant hits from the GWAS by Wootton and colleagues (1), and represents a weakly heritable trait. Likewise, the confounding effect of 6 was chosen so that the effect of the confounders was much stronger than the true effect. Since the effect of weak-instrument bias is bounded by size of the confounding effect, choosing a large value for the confounder should make the simulation adversarial in settings with weak-instrument bias. Indeed, confounding explains around 90% of the variation in PP1 and PP2. As such, these phonotypes are majority environmentally determined.

We then simulated the offspring’s phenotype in four settings:

A causal effect and no assortative mating.

In this setting we simulated the child’s phenotype when the parental phenotypes impact on it (i.e., under the alternative) as:

CPA = CGR + C1*6 + C2*6 + 0.75*PP1 + 0.75*PP2 + ε_3_

ε_3_ is an error term simulated as independent and identically distributed normal distributions such that ε_3_ ~ N(0, 1^2^). The true standardised effect under the alternative of 0.75 was chosen to represent the large expected effect between parental and offspring smoking (3). Each PP1 and PP2 have a partial r^2^ on the CPA, conditional on C1 and C2, of around 6%. The partial r^2^ of CGR [the child’s genetic risk score] conditional on P1GR and P2GR is, as with the parents, about 0.5%. C1 and C2 together explain over 90% of the variation in CPA. As such, despite the large size of effect that the parental phenotype exerts, the child’s phenotype is mostly determined by other environmental factors and not parental or genetic effects – as is arguably common among many psychological and behavioural traits (4).

The study population was initially simulated with 13,600 offspring-parent trios. In the no assortative mating setting, we analysed a random 59% of this sample. This was intended to result in samples of around 10,000 trios, as an approximate estimate of the sample available in ALSPAC without any loss-to-follow up.

A causal effect and assortative mating.

In the setting with a causal effect and assortative mating we simulated CPA as in the “causal effect and no assortative mating” setting. However, in this setting we selected trios whose second parent had a genetic liability to the exposure within 0.9 standard deviations of the standardised genetic liability of the first parent. This was chosen to represent assortative mating resulting in a parental genetic correlation of around 0.65 and thus stress test the method in a setting with a slightly stronger genetic correlation than what has thus far been observed in reality for smoking (5).

The initial sample size of 13,600 trios was chosen so that this selection process would again result in samples of around 10,000 trios. To explore how Proxy-MVMR-NIV performs with stronger instruments, we repeated the simulation for this approach in a sample with ten times more parent-child trios, in order to simulate a sample with 100,000 parent-child duos to represent the sample size that is available from samples like The Norwegian Mother, Father and Child Cohort Study (MoBa) (6).

No causal effect and no assortative mating

In this setting, the child’s phenotype was simulated under the null as:

CPN = CGR + C1*6 + C2*6 + ε_4_

ε_4_ is an error term simulated as independent and identically distributed normal distributions such that ε_4_ ~ N(0, 1^2^). The final study population was again derived by randomly sampling 59% of the 13,600 trios originally simulated. Under the null hypothesis, again around 0.5% of variation is explained by the child’s genetic risk and >90% by environmental confounding.

No causal effect and assortative mating

In the setting with no casual effect and no assortative mating, CPN was simulated the same as in the setting without assortative mating. Assortative mating was induced using an identical approach to that described for the “causal effect and assortative mating” setting. We implemented this simulation with the same two sample sizes used in the simulation with a causal effect and assortative mating to again represent an ALSPAC and MOBA sized sample.

Changes to some of the variables’ values could change the precise numerical results of the simulation. To improve generalisability, we will therefore quickly characterize how changing some of the key parameters should change the simulation results in terms of power, weak instrument bias, and bias in univariable models due to assortative mating:

- Decreasing the size of genetic effects will: reduce power, increase weak instrument bias, reduce the absolute magnitude of bias due to assortative mating (although assortative mating can take many forms and this may not hold in all applied settings).
- Reducing the error variance in the parental phenotype will reduce weak instrument bias, but should not impact on power or bias due to assortative mating all else being equal.
- Increasing the effect of confounding will increase weak instrument bias. It may also reduce power by increasing the residual phenotypic variation. Since we modelled each parent as having a unique confounder, it should not impact on bias from assortative mating.
- Increasing the parental effect will increase power, decreasing it will decrease power. It should not, however, change the amount of weak instrument bias or bias due to assortative mating.
- Increasing error variance in the offspring phenotype will reduce power. It should not impact on weak instrument bias or bias due to assortative mating.
- Increasing the correlation between parents when there is assortative mating should increase bias (in univariable models), decreasing it should decrease bias (in univariable models) due to assortative mating. All else being equal, it should not impact on power or weak instrument bias.”

*1.2. Further details on the Simulation Methods*

We explore the MR-NIV estimators described in Table 1:

1. Using the actual non-inherited variants from a single parent as an instrument for that parent’s phenotype in models that additionally adjust for the child’s genetic risk score.
2. Using the actual non-inherited variants from both parents as instruments for both parent’s phenotypes in models that additionally adjust for the child’s genetic risk score.
3. Using a single parent’s genetic risk score (with inherited and non-inherited variants) as an instrument for that parent’s phenotype in models that additionally adjust for the child’s genetic risk score.
4. Using both parents’ genetic risk (with inherited and non-inherited variants) as instruments for both parent’s phenotypes in models that additionally adjust for the child’s genetic risk score.
5. Using the actual non-inherited variants from both parents as instruments for a linear combination of both parent’s phenotypes in models that additionally adjust for the child’s genetic risk score.
6. Using both parents’ parental genetic risk as instruments for a linear combination of both parent’s phenotypes in models that additionally adjust for the child’s genetic risk score.

These were all implemented using two-stage least square regression. The creation of inherited/non-inherited risk scores is described in the ‘Inferring inherited and non-inherited alleles from parental and offspring allele scores’ section below.

In settings with assortative mating, we also explored the performance of Proxy-MVMR-NIV for addressing AM when there is only genetic information from one parent. Specifically, using the actual non-inherited variants from one single parent as instruments for that parent’s phenotype, and the inherited variants from the other parent as instruments for that parent’s phenotype in models which additionally adjust for the child’s genetic risk score. Because of expected weak-instrument bias, we only implemented Proxy-MVMR-NIV using weak-instrument robust estimators: GMM, debiased IVW, GRAPPLE, and QHet (7–10). These estimators are described in a later section. We also note that we only implement Proxy-MVMR-NIV in the MOBA sized simulations.

2. Applied Example in ALSPAC

*2.1. The Avon Longitudinal Study of Parents and Children (ALSPAC) sample description*

Pregnant women resident in Avon, UK with expected dates of delivery between 1st April 1991 and 31st December 1992 were invited to take part in the study (11–13). 20,248 pregnancies have been identified as being eligible and the initial number of pregnancies enrolled was 14,541. Of the initial pregnancies, there was a total of 14,676 fetuses, resulting in 14,062 live births and 13,988 children who were alive at 1 year of age. When the oldest children were approximately 7 years of age, an attempt was made to bolster the initial sample with eligible cases who had failed to join the study originally. As a result, when considering variables collected from the age of seven onwards (and potentially abstracted from obstetric notes) there are data available for more than the 14,541 pregnancies mentioned above: The number of new pregnancies not in the initial sample (known as Phase I enrolment) that are currently represented in the released data and reflecting enrolment status at the age of 24 is 906, resulting in an additional 913 children being enrolled (456, 262 and 195 recruited during Phases II, III and IV respectively).

The phases of enrolment are described in more detail in the cohort profile paper and its update (11,12). The total sample size for analyses using any data collected after the age of 7 is therefore 15,447 pregnancies, resulting in 15,658 foetuses. Of these 14,901 children were alive at 1 year of age. Of the original 14,541 initial pregnancies, 338 were from a woman who had already enrolled with a previous pregnancy, meaning 14,203 unique mothers were initially enrolled in the study. As a result of the additional phases of recruitment, a further 630 women who did not enrol originally have provided data since their child was 7 years of age. This provides a total of 14,833 unique women (G0 mothers) enrolled in ALSPAC as of September 2021.

G0 partners were invited to complete questionnaires by the mothers at the start of the study and they were not formally enrolled at that time. 12,113 G0 partners have been in contact with the study by providing data and/or formally enrolling when this started in 2010. 3,807 G0 partners are currently enrolled.

*2..2 Measures*

*Outcome*: Offspring reported current smoking status at age 16 years (ALSPAC variable name = ccs4005). The (offspring) participants were asked how frequently they smoke (given that they have smoked at least once) with options of: [1] once or twice, [2] used to smoke but no longer, [3] less than once a week, [4] one to six cigarettes a week, [5] more than 6 cigarettes a week but not daily, [6] smokes daily. We defined being a current smoker as a participant having given response [3] or higher.

*Exposures*: Maternal reported number of cigarettes smoked per day by the mother (s1301 and s1300) and partner (s3070 and s3071) when the child was 12. This was chosen as the nearest time point for when parental smoking was ascertained prior to measurement of offspring smoking at 16 years. We estimated the number of packs smoked per week as: 5 times the number of cigarettes smoked per day on a weekday plus two times the number of cigarettes smoked per weekend day, all divided by 20.

*Other covariates for the substantive MR-NIV model*: First 10 principal components of ancestry for the child and each parent included in the model; Offspring sex assigned at birth (kz021); the age of each parent included in the model at delivery of the child (mz028b and partner_age for maternal and paternal age respectively).

*Additional covariates for the observational analysis:* In addition to the covariates used in the MR-NIV model, the observation analysis adjusted for additional parental covariates: highest educational qualification (c666a, c686a), socioeconomic position at recruitment (c755, c765, b_seg_m, b_sc-m, b_empst_m, pb_empst_p, pb_sc_p, and pb_seg_p).

*Positive controls:* Maternal reported high blood pressure for the mother (s1016) and partner (s3029) when the child was aged 12 years.

*Additional auxiliary variables for imputation*: In addition to the variables already described, we used the following variables as auxiliary variables for the imputation model based on the covariate choice of existing studies which used multiple imputation (14–16). Offspring covariates: self-reported smoking at 14 years (ccr705). Parental covariates: number of cigarettes smoked per day in the past two weeks by the mother and partner when the study child was aged 1 and 8 years (g820, g648, n5010, n5042) as reported by the mother.

*2.3. Statistical analysis plan*

*Primary analysis:* The primary aim of the applied example is to use ALSPAC to assess the causal effect of parental genetically predicted smoking on their offspring’s current smoking status age 16. Specifically, we looked at the effect of parental genetically predicted lifetime smoking (scaled to the effect of an additional pack of cigarette smoked per week when the offspring was aged 12) on offspring current smoking at age 16.

We split our dataset into three: families with trio data, families without trio data but maternal genetics, and families without trio data but paternal genetics. For those without trio data we ran UVMR-NIV models. Because we have a binary outcome, these were estimated using the Wald ratios by regressing offspring smoking on the GRS using logistic regression, and then dividing this by the estimate from a regression of the parents smoking on the GRS. To maximise precision, the trio analysis used a composite of both parent’s smoking as the exposure. Both the first and second stage regression models adjusted for the phenotypes described in the previous section. Rather than regressing this on each parent’s GRSs separately, to simplify the statistical model we combined both parents non-inherited alleles into a single risk score. This approach makes similar assumptions (i.e. linearity and additivity) to modelling both parent’s GRSs separately but is computationally easier to implement for Wald ratios. For families where genetic information was available on only one parent, only that parent’s smoking and GRS were included in the model. We also conducted a crude (i.e. unadjusted for covariates) observational analyses, as well as an analysis adjusted for the covariates described in the *Measures* sub-section above. Please note that parental covariates were only included in models for which that parent’s smoking was included as an exposure.

To minimise risk of bias from population structure, we restricted our sample to participants whose parents’ self-reported as of European ancestry (c804). We additionally exclude partners who the mother did not report as the father of the child (a521).

*Multiple imputation:* We address phenotypic missing data (but not missing genotypes) using multiple imputation. The imputation model included all variables included in the substantive models as well as various auxiliary variables described in the *Measures* sub-section above. The imputation process was implemented using Multiple Imputation with Chained Equations (MICE) with 25 imputed datasets and 25 iterations in the chained equations. For simplicity, rather than including each SNP in the imputation model we included the offspring GRS and the two parental non-inherited GRSs. Under the assumptions of linearity and additivity made by our substantive models, this should be equivalent to including each SNP or including the two parental GRSs. We also ran a complete case analysis with the imputed data.

*Positive control outcome:* Smoking is known to impact on blood pressure (17). We use each maternal reported parental hypertension at the same time point as when parental smoking was measured (child aged 12) as a positive control outcome.

*MR sensitivity analyses:* We assess weak-instrument bias using first stage F-statistics the MR models, and (for the primary MR analysis) used the three weak-instrument robust methods described in the Weak-Instrument Robust Estimators section of Supplementary Methods in secondary analyses which can be used in a univariable MR setting (7–10). The three weak-instrument robust estimators used here are all summary data-based estimators which require multiple instruments. Rather than using Wald ratios derived from weighted GRS, we therefore implement them by regressing the exposure and then the outcome on each non-inherited SNP. Finally, we assess risk of bias due to pleiotropy by examining the I^2^ statistic for the MR Wald ratio of each SNP in the primary analyses (18).

3. Weak-Instrument Robust Estimators

In this paper we use four so called ‘weak-instrument robust’ estimators:

The first is the Continuously updating Generalized Method of Moments (GMM) estimator (18), a well established and popular method to estimate IV models. This method has been shown theoretically (19), and in simulation (20) to guard against weak instrument bias under “many weak instrument” asymptotics. Moreover, these robustness properties extend to our summary data setting in linear IV models, as shown theoretically in Theorems 3.1 and 3.2 of Zhao and colleagues (21). We further note that the limited information maximum likelihood (LIML) estimator (22) is a special case of the continuously updating GMM estimator in a linear IV model with homoscedastic errors (23). We implemented the continuously updating GMM method using the MendelianRandomization R package (24,25).

The second estimator is QHet, which is the oldest weak-instrument estimator specifically introduced for use in multivariable MR analyses (7). QHet attempts to mitigate weak-instrument bias through an algorithm which minimising the between SNP heterogeneity. This additionally provides QHet with some robustness to balanced pleiotropy. Unlike the other weak-instrument methods used here, QHet was not designed to work in a ‘one sample’ IV setting. It therefore assumes that errors in the variant-exposure estimates are uncorrelated to errors in the variant-outcome estimates. Please note that since there is no univariable MR analogue of QHetT we do not implement this estimator in univariable MR analyses.

The third estimator is Genome-wide mR Analysis under Pervasive PLEiotropy (GRAPPLE) (10). This estimator can be thought of as a multivariable implementation of the widely used MR-RAPS estimator (22). Both approaches use profile maximum-likelihood maximisation in an attempt to minimise weak-instrument bias. We use MR-RAPS (with a Tukey loss function and no over dispersion) in univariable MR analyses.

The final estimator is Debiased IVW (9). The debiased IVW estimator is a modified version of the traditional IVW estimator which uses theoretically derived bias equations to analytically account for weak-instrument bias, and thereby improve the asymptotic properties of the IVW estimator. Although both GRAPPLE and Debiased IVW have been designed for use in ‘two-sample MR’ analyses, both can account for sample overlap through specifying the error correlation matrix between relevant traits.

**Supplementary Table S1: Results of simulation when the parent’s phenotype does cause the child’s phenotype. Standard errors are Monte-Carlo standard errors.**

| **Assortative Mating** | **Outcome** | **Parent** | **UVMR-NIV** | **UVMR adjusted for child GRS** | **MVMR-NIV** | **MVMR adjusted for child GRS** |
| --- | --- | --- | --- | --- | --- | --- |
| With no assortative mating | Bias (SE MCSE) | Parent 1 | 0.002 (0.002) | -0.239 (0.003) | -0.005 (0.002) | -0.004 (0.001) |
|  |  | Parent 2 | 0.002 (0.002) | -0.239 (0.003) | -0.005 (0.002) | -0.004 (0.001) |
|  | Coverage (MCSE) | Parent 1 | 0.952 (0.007) | 0.336 (0.015) | 0.954 (0.007) | 0.955 (0.007) |
|  |  | Parent 2 | 0.947 (0.007) | 0.32 (0.015) | 0.952 (0.007) | 0.949 (0.007) |
|  | 95% CI width (MCSE) | Parent 1 | 0.462 (0.001) | 0.4 (0.001) | 0.326 (0.001) | 0.28 (0.001) |
|  |  | Parent 2 | 0.463 (0.001) | 0.401 (0.001) | 0.326 (0.001) | 0.28 (0.001) |
|  | Variance of beta across simulations | Parent 1 | 0.015 | 0.010 | 0.007 | 0.005 |
|  |  | Parent 2 | 0.014 | 0.010 | 0.007 | 0.005 |
|  | F-statistic | Parent 1 | 284 | 433 | 275 | 356 |
|  |  | Parent 2 | 283 | 432 | 272 | 353 |
| With assortative mating | Bias (MCSE) | Parent 1 | 0.501 (0.005) | 0.331 (0.004) | -0.003 (0.004) | 0 (0.002) |
|  |  | Parent 2 | 0.503 (0.005) | 0.329 (0.004) | -0.001 (0.004) | -0.004 (0.002) |
|  | Coverage (MCSE) | Parent 1 | 0.136 (0.011) | 0.302 (0.015) | 0.969 (0.005) | 0.945 (0.007) |
|  |  | Parent 2 | 0.144 (0.011) | 0.3 (0.014) | 0.976 (0.005) | 0.958 (0.006) |
|  | 95% CI width (MCSE) | Parent 1 | 0.628 (0.002) | 0.513 (0.001) | 0.798 (0.006) | 0.435 (0.002) |
|  |  | Parent 2 | 0.628 (0.002) | 0.514 (0.001) | 0.798 (0.006) | 0.435 (0.002) |
|  | Variance of beta across simulations | Parent 1 | 0.027 | 0.016 | 0.041 | 0.012 |
|  |  | Parent 2 | 0.026 | 0.017 | 0.038 | 0.012 |
|  | F-statistic | Parent 1 | 130 | 204 | 33 | 137 |
|  |  | Parent 2 | 129 | 203 | 33 | 135 |

MCSE = Monte Carlo Standard Error.

**Supplementary Table S2: Results of simulation when the parent’s phenotype does not cause the child’s phenotype. Standard errors are Monte-Carlo standard errors.**

| **Assortative mating** | **Outcome** | **Parent** | **UVMR-NIV** | **UVMR adjusted for child GRS** | **MVMR-NIV** | **MVMR adjusted for child GRS** |
| --- | --- | --- | --- | --- | --- | --- |
| With no assortative mating | Bias (MCSE) | Parent 1 | -0.004 (0.002) | -0.002 (0.001) | -0.005 (0.002) | -0.004 (0.001) |
|  |  | Parent 2 | -0.005 (0.002) | -0.002 (0.001) | -0.005 (0.002) | -0.004 (0.001) |
|  |  | Mean of Parrent 1 and Parrent 2 | -0.005 | -0.002 | -0.005 | -0.004 |
|  | Coverage (MCSE) | Parent 1 | 0.952 (0.007) | 0.958 (0.006) | 0.954 (0.007) | 0.959 (0.006) |
|  |  | Parent 2 | 0.950 (0.007) | 0.953 (0.007) | 0.952 (0.007) | 0.948 (0.007) |
|  |  | Mean of Parrent 1 and Parrent 2 | 0.951 | 0.9566 | 0.953 | 0.954 |
|  | 95% CI width (MCSE) | Parent 1 | 0.324 (0.001) | 0.264 (0.001) | 0.326 (0.001) | 0.280 (0.001) |
|  |  | Parent 2 | 0.325 (0.001) | 0.264 (0.001) | 0.326 (0.001) | 0.280 (0.001) |
|  |  | Mean of Parrent 1 and Parrent 2 | 0.325 | 0.264 | 0.326 | 0.280 |
|  | Variance of beta across simulations | Parent 1 | 0.007 | 0.004 | 0.007 | 0.005 |
|  |  | Parent 2 | 0.007 | 0.004 | 0.007 | 0.005 |
|  |  | Mean of Parrent 1 and Parrent 2 | 0.007 | 0.004 | 0.007 | 0.005 |
| With assortative mating | Bias (MCSE) | Parent 1 | -0.005 (0.003) | -0.002 (0.002) | -0.003 (0.004) | -0.001 (0.002) |
|  |  | Parent 2 | -0.003 (0.002) | -0.003 (0.002) | 0.000 (0.004) | -0.003 (0.002) |
|  |  | Mean of Parrent 1 and Parrent 2 | -0.004 | -0.003 | -0.002 | -0.002 |
|  | Coverage (MCSE) | Parent 1 | 0.943 (0.007) | 0.940 (0.008) | 0.969 (0.005) | 0.949 (0.007) |
|  |  | Parent 2 | 0.954 (0.007) | 0.949 (0.007) | 0.971 (0.005) | 0.952 (0.007) |
|  |  | Mean of Parrent 1 and Parrent 2 | 0.949 | 0.945 | 0.970 | 0.951 |
|  | 95% CI width (MCSE) | Parent 1 | 0.488 (0.002) | 0.387 (0.001) | 0.798 (0.006) | 0.435 (0.002) |
|  |  | Parent 2 | 0.487 (0.002) | 0.388 (0.001) | 0.798 (0.006) | 0.435 (0.002) |
|  |  | Mean of Parrent 1 and Parrent 2 | 0.488 | 0.388 | 0.798 | 0.435 |
|  | Variance of beta across simulations | Parent 1 | 0.016 | 0.010 | 0.040 | 0.012 |
|  |  | Parent 2 | 0.015 | 0.010 | 0.039 | 0.012 |
|  |  | Mean of Parrent 1 and Parrent 2 | 0.016 | 0.010 | 0.040 | 0.012 |

MCSE = Monte Carlo Standard Error. Since the F-statistics in this table were the same as the ones presented in Supplementary Table S2, for simplicity we have only presented the F-statistics in that table. For the purpose of comparisons to Table 2, we have added the average of each parent’s score to this table (but not to Supplementary Table S2).

**Supplementary Table S3: Results of MVMR-NIV using a composite parental exposure**

| **Assortative mating** | **Causal effect is** | **Outcome** | **Bias (MCSE)** | **Coverage (MCSE)** | **95% CI width (MCSE)** | **Variance of beta across simulations** |
| --- | --- | --- | --- | --- | --- | --- |
| With no assortative mating | Null | MVMR-NIV | -0.001 (0.001) | 0.947 (< 0.000) | 0.251 (0.001) | 0.004 |
|  |  | MVMR adjusted for child GRS | -0.001 (0.001) | 0.949 (< 0.000) | 0.226 (0.001) | 0.003 |
|  | Non-null | MVMR-NIV | -0.002 (0.001) | 0.942 (0.007) | 0.251 (0.001) | 0.004 |
|  |  | MVMR adjusted for child GRS | -0.001 (0.001) | 0.946 (0.007) | 0.226 (0. 001) | 0.003 |
| With assortative mating | Null | MVMR-NIV | 0.002 (0.001) | 0.939 (< 0.000) | 0.252 (0.001) | 0.004 |
|  |  | MVMR adjusted for child GRS | 0.001 (0.001) | 0.947 (< 0.000) | 0.227 (0.001) | 0.003 |
|  | Non-null | MVMR-NIV | 0.001 (0.001) | 0.939 (0.008) | 0.252 (0.001) | 0.004 |
|  |  | MVMR adjusted for child GRS | 0.001 (0.001) | 0.946 (0.007) | 0.227 (0.001) | 0.003 |

MCSE = Monte Carlo Standard Error.

**Supplementary Table S4: Results of Proxy-MVMR-NIV in settings with assortative mating in an ALSPAC scale sample.**

| **Estimator** | **QHet** | | **GMM** | | **Debiased IVW** | | **GRAPPLE** | |
| --- | --- | --- | --- | --- | --- | --- | --- | --- |
| Causal effect is | Non-null | Null | Non-null | Null | Non-null | Null | Non-null | Null |
| Parent 1 Bias (MCSE) | 0.638 (0.002) | 0.637 (0.002) | 0.623 (0.001) | 0.631 (0.002) | -0.097 (0.109) | -0.008 (0.122) | -0.641 (0.169) | 0.498 (0.121) |
| Parent 2 Bias (MCSE) | 0.633 (0.002) | 0.633 (0.002) | 0.622 (0.001) | 0.628 (0.002) | 0.068 (0.187) | 0.198 (0.184) | -0.916 (0.163) | 0.345 (0.113) |
| Parent 1 Coverage (MCSE) | NA | NA | 0 (< 0.000) | 0 (< 0.000) | 0.964 (0.006) | 0.973 (0.005) | 0.989 (0.003) | 0.989 (0.003) |
| Parent 2 Coverage (MCSE) | NA | NA | 0 (< 0.000) | 0 (< 0.000) | 0.964 (0.006) | 0.971 (0.005) | 0.995 (0.002) | 0.995 (0.002) |
| Parent 1 95% CI width (MCSE) | NA | NA | 0.688 (0.001) | 0.371 (0.001) | 163.009 (130.917) | 178.182 (163.098) | 3640.506 (1746.359) | 641.947 (133.517) |
| Parent 2 95% CI width (MCSE) | NA | NA | 0.69 (0.001) | 0.372 (0.001) | 176.232 (86.697) | 159.957 (97.643) | 4817.351 (4091.329) | 1410.434 (763.939) |
| Parent 1 Variance of beta across simulations | 0.005 | 0.005 | 0.002 | 0.003 | 12.499 | 15.107 | 33.638 | 16.871 |
| Parent 2 Variance of beta across simulations | 0.004 | 0.004 | 0.002 | 0.003 | 36.02 | 34.174 | 31.113 | 14.837 |

The mean conditional F-statistics for both Parent 1 and Parent 2 in this simulation was 3. MCSE = Monte Carlo Standard Error.

**Supplementary Table S5: Results of Proxy-MVMR-NIV in settings with assortative mating in an MoBa scale sample.**

| **Estimator** | **QHet** | | **GMM** | | **Debiased IVW** | | **GRAPPLE** | |
| --- | --- | --- | --- | --- | --- | --- | --- | --- |
| Causal effect is | Non-null | Null | Non-null | Null | Non-null | Null | Non-null | Null |
| Parent 1 Bias (MCSE) | 0.072 (0.003) | 0.065 (0.002) | 0.046 (0.002) | 0.024 (0.001) | -0.333 (0.419) | -0.536 (0.46) | -0.048 (0.062) | 0.249 (0.051) |
| Parent 2 Bias (MCSE) | 0.075 (0.003) | 0.065 (0.002) | 0.048 (0.002) | 0.022 (0.002) | 1.12 (2.924) | 1.482 (3.401) | -0.08 (0.062) | 0.161 (0.053) |
| Parent 1 Coverage (MCSE) | NA | NA | 1 (< 0.000) | 0.877 (0.010) | 0.793 (0.013) | 0.989 (0.003) | 0.973 (0.005) | 0.976 (0.005) |
| Parent 2 Coverage (MCSE) | NA | NA | 0.999 (0.001) | 0.874 (0.010) | 0.784 (0.013) | 0.985 (0.004) | 0.969 (0.005) | 0.966 (0.006) |
| Parent 1 95% CI width (MCSE) | NA | NA | 0.41 (0.001) | 0.215 (< 0.000) | 1350.318 (1242.962) | 1456.727 (1392.440) | 178.603 (61.772) | 155.937 (85.772) |
| Parent 2 95% CI width (MCSE) | NA | NA | 0.41 (0.001) | 0.214 (< 0.000) | 220396.757 (201360.11) | 237410.451 (217976.657) | 152.986 (41.757) | 73.509 (21.373) |
| Parent 1 Variance of beta across simulations | 0.007 | 0.007 | 0.002 | 0.005 | 176.978 | 212.096 | 4.619 | 3.003 |
| Parent 2 Variance of beta across simulations | 0.007 | 0.007 | 0.002 | 0.005 | 8567.714 | 11587.5 | 4.57 | 3.184 |

The mean conditional F-statistics was 17 for Parrent 1 and 18 for Parent 2 in this simulation. MCSE = Monte Carlo Standard Error.

**Supplementary Table S6: Algorithm used to decide if an allele was inherited or not.**

| Condition | Number of P1 non-inherited effect allele | Number of P2 inherited effect allele |
| --- | --- | --- |
| P1 has 2 effect alleles | 1 | Number of effect alleles that the C has minus one |
| P1 has zero effect alleles | 0 | Number of effect allele’s that the C has |
| P1 has 1 effect allele and C has 2 effect alleles | 0 | 1 |
| P1 has 1 effect allele and C has 0 effect alleles | 1 | 0 |
| P1 has 1 effect allele, C has 1 effect alleles and we have not measured P2’s genotype | NA* | NA* |
| P1 has 1 effect allele, C has 1 effect alleles and P2 has 0 effect allele | 0 | 0 |
| P1 has 1 effect allele, C has 1 effect alleles and P2 has 2 effect allele | 1 | 1 |
| P1 has 1 effect allele, C has 1 effect alleles and P2 has 1 effect allele | NA* | NA* |

* In this setting, it is unknown if the effect allele was inherited or not. In this case, we exclude the variant from the relevant risk scores. This is to minimise risk of a potential exclusion restriction violation from including an actually inherited allele.

N.B. This algorithm was developed for a setting in which the number of effect alleles of each variant for at least one parent and offspring is known. The algorithm can then be used to determine the number of alleles that should be included in the non-inherited or inherited (effect) allele GRS for each variant. It requires assuming that there is a very low measurement error in the genotyping. It is therefore not suitable for variants with, e.g., a low INFO score (23). New mutations imply that the offspring did not inherit the allele from their parents, and therefore is unlikely to bias this algorithm.

**Supplementary Table S7: Descriptive results from ALSPAC**

| **phenotype** | **N with Observations** | **N Missing** | **Data Type** | **Mean (SD) or N exposed (%)** | **Effect on child smoking at 16, crude OR (95% CI)*** |
| --- | --- | --- | --- | --- | --- |
| Child smoking status at age 16 (smoker) | 3349 | 4745 | factor | 637 (19.0) | NA |
| Number of packs smoked per week by the mother when the child was aged 12 | 4325 | 3769 | numeric | 0.576 (1.702) | 1.124 (1.068 - 1.184) |
| Number of packs smoked per week by the father when the child was aged 12 | 3974 | 4120 | numeric | 0.823 (2.363) | 1.107 (1.066 - 1.149) |
| Child sex (Male) | 6886 | 1208 | factor | 3469 (50.4) | 0.555 (0.462 - 0.667) |
| Mother’s age when the child was born | 6886 | 1208 | integer | 28.889 (4.577) | 1.001 (0.981 - 1.021) |
| Father’s age when the child was born | 6299 | 1795 | integer | 31.289 (5.561) | 1 (0.984 - 1.016) |
| Maternal hypertension | 4373 | 3721 | factor | 269 (6.2) | 0.985 (0.663 - 1.462) |
| Paternal hypertension | 3592 | 4502 | factor | 399 (11.1) | 0.864 (0.613 - 1.219) |
| Number of cigarettes smoked every two weeks by the mother when the child was aged 8. | 4888 | 3206 | numeric | 1.958 (5.433) | 1.041 (1.024 - 1.058) |
| Number of cigarettes smoked per day by the father when the child was aged 8. | 4887 | 3207 | numeric | 2.395 (6.204) | 1.034 (1.019 - 1.049) |
| Number of cigarettes smoked per day by the mother when the child was aged 1. | 5886 | 2208 | integer | 1.957 (5.128) | *1.04 (1.022 - 1.059) |
| Number of cigarettes smoked per day by the father when the child was aged 1. | 5659 | 2435 | integer | 3.177 (7.044) | 1.034 (1.021 - 1.047) |
| Frequency that the child smoked when aged 14 | 3872 | 4222 | numeric | 0.463 (1.090) | 2.28 (2.052 - 2.533) |
| Maternal education level when the child was aged 1* | 5204 | 2890 | factor | 2.223 (1.307) | 1.059 (0.985 - 1.138) |
| Maternal class when the child was born* | 6039 | 2055 | factor | 1899 (31.4) | 1.218 (0.792 - 1.875) |
| Maternal socio-economic position when the child was born | 6039 | 2055 | numeric | 6.147 (2.089) | 1.068 (1.02 - 1.117) |
| Maternal employment when the child was born* | 6043 | 2051 | factor | 4.815 (0.558) | 1.019 (0.868 - 1.197) |
| Paternal class when the child was born* | 5418 | 2676 | factor | 2.966 (1.235) | 1.094 (1.012 - 1.183) |
| Paternal socio-economic position when the child was born | 5418 | 2676 | numeric | 6.523 (3.127) | 1.036 (1.004 - 1.07) |
| Paternal employment when the child was born* | 5418 | 2676 | factor | 3.933 (1.158) | 0.906 (0.834 - 0.983) |

* Numerical values for the categorical socio-economic variables should not be interpreted.

**Supplementary Table S8: Heterogeneity (I^2^ statistics) for the SNP specific Wald ratios for the primary MR analyses**

|  | Not imputed data sets | Imputed data sets |
| --- | --- | --- |
| Mothers without trio data | 0.291 | 0.000 |
| Fathers without trio data | 0.000 | 0.000 |
| Mothers and Fathers with trio data | 0.000 | 0.000 |

**Supplementary Figure S1: Weak-instrument robust estimators for the primary analysis in ALSPAC**

| **Estimator** | **Using imputed data** | **Using non-imputed data** |
| --- | --- | --- |
| **PC-GMM** | **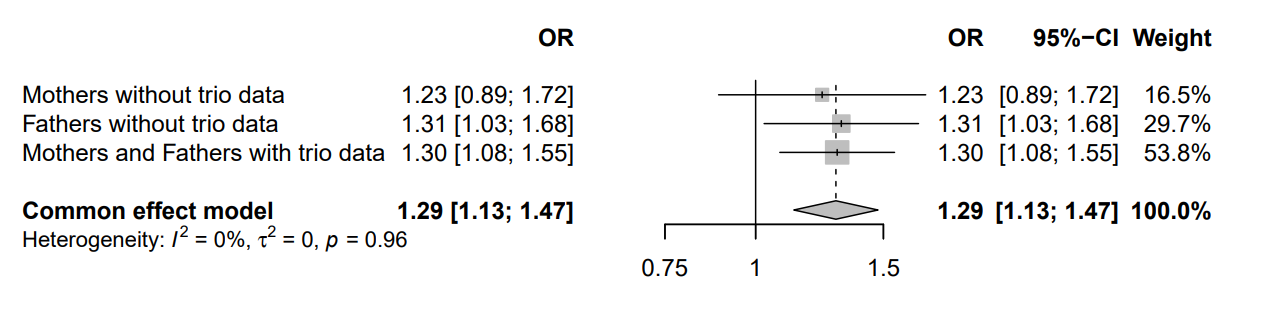** | **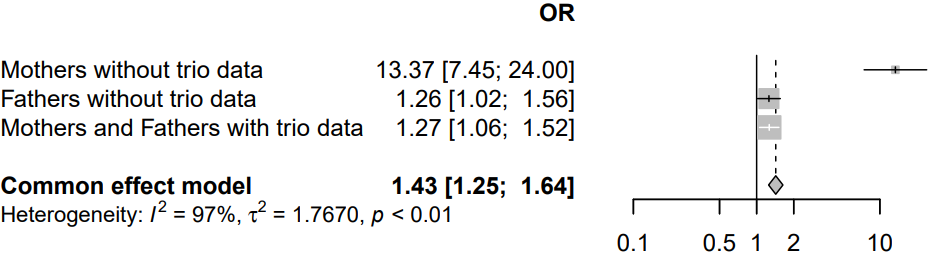** |
| **RAPS** | **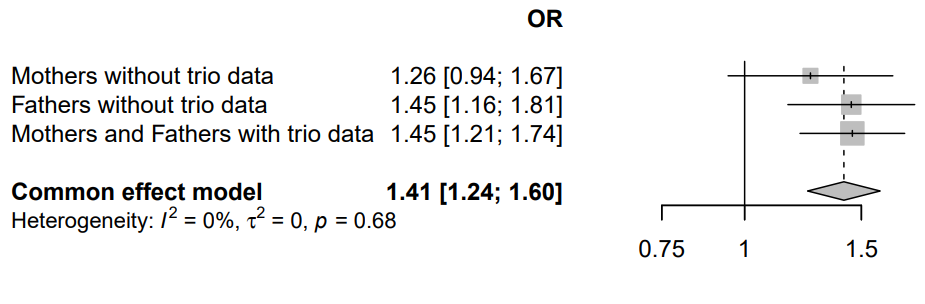** | **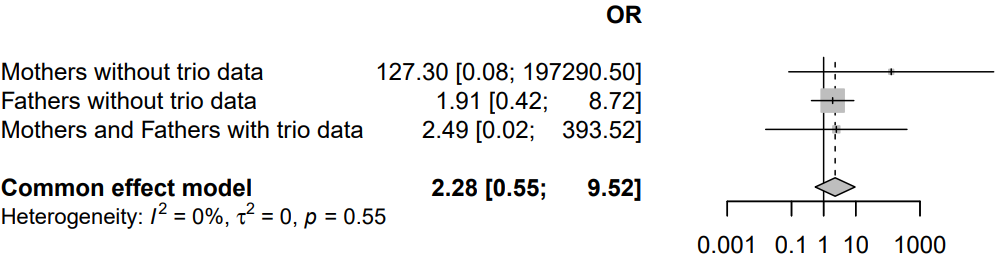** |
| **Debiased IVW** | **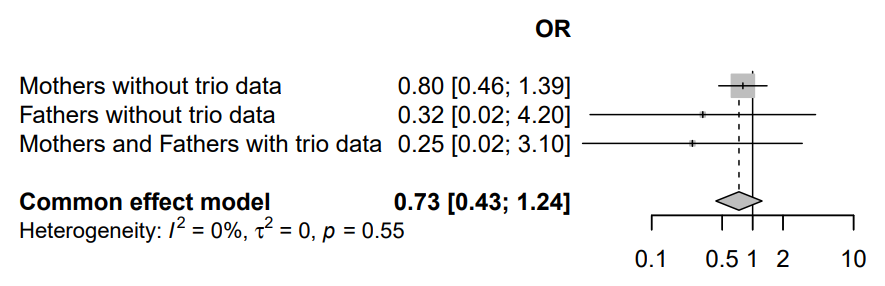** | **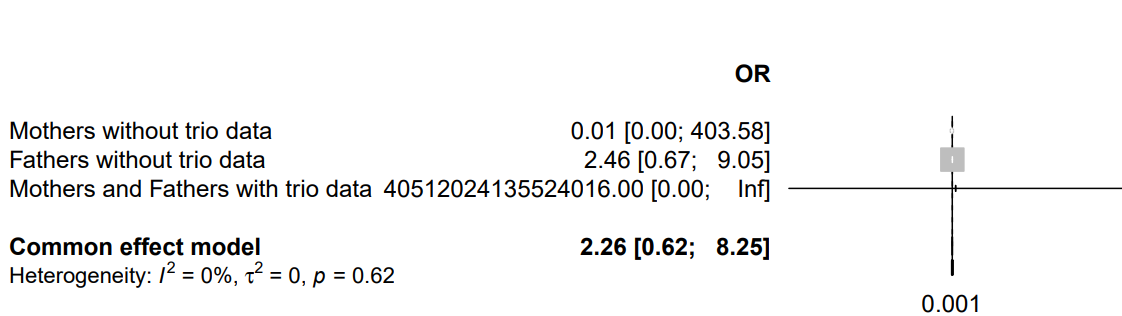** |

ORs represent the multiplicative increase in the odds of the child smoking at age 16 per genetically predicted pack smoked by a parent when the child was aged 12.

**References**

1. Wootton RE, Richmond RC, Stuijfzand BG, Lawn RB, Sallis HM, Taylor GMJ, et al. Evidence for causal effects of lifetime smoking on risk for depression and schizophrenia: a Mendelian randomisation study. Psychol Med. 2020 Oct;50(14):2435–43.

2. Evans DM, Smith GD, Moen GH. Woolf et al’s “GWAS by subtraction” is not useful for cross-generational Mendelian randomization studies. BMC Res Notes. 2025 Jun 26;18(1):247.

3. Alves J, Perelman J, Soto-Rojas V, Richter M, Rimpelä A, Loureiro I, et al. The role of parental smoking on adolescent smoking and its social patterning: a cross-sectional survey in six European cities. Journal of Public Health. 2017 Jun 1;39(2):339–46.

4. Harris JR. Where is the child’s environment? A group socialization theory of development. Psychological Review. 1995;102(3):458–89.

5. Agrawal A, Heath AC, Grant JD, Pergadia ML, Statham DJ, Bucholz KK, et al. Assortative mating for cigarette smoking and for alcohol consumption in female Australian twins and their spouses. Behav Genet. 2006 Jul;36(4):553–66.

6. Magnus P, Birke C, Vejrup K, Haugan A, Alsaker E, Daltveit AK, et al. Cohort Profile Update: The Norwegian Mother and Child Cohort Study (MoBa). Int J Epidemiol. 2016 Apr 1;45(2):382–8.

7. Sanderson E, Spiller W, Bowden J. Testing and correcting for weak and pleiotropic instruments in two-sample multivariable Mendelian randomization. Statistics in Medicine. 2021;40(25):5434–52.

8. Burgess S, Small DS, Thompson SG. A review of instrumental variable estimators for Mendelian randomization. Stat Methods Med Res. 2017 Oct;26(5):2333–55.

9. Wu Y, Kang H, Ye T. Debiased Multivariable Mendelian Randomization [Internet]. arXiv; 2024 [cited 2024 Jul 23]. Available from: http://arxiv.org/abs/2402.00307

10. Wang J, Zhao Q, Bowden J, Hemani G, Smith GD, Small DS, et al. Causal inference for heritable phenotypic risk factors using heterogeneous genetic instruments. PLOS Genetics. 2021 Jun 22;17(6):e1009575.

11. Boyd A, Golding J, Macleod J, Lawlor DA, Fraser A, Henderson J, et al. Cohort Profile: the ’children of the 90s’--the index offspring of the Avon Longitudinal Study of Parents and Children. Int J Epidemiol. 2013 Feb;42(1):111–27.

12. Fraser A, Macdonald-Wallis C, Tilling K, Boyd A, Golding J, Davey Smith G, et al. Cohort Profile: the Avon Longitudinal Study of Parents and Children: ALSPAC mothers cohort. Int J Epidemiol. 2013 Feb;42(1):97–110.

13. Northstone K, Ben Shlomo Y, Teyhan A, Hill A, Groom A, Mumme M, et al. The Avon Longitudinal Study of Parents and children ALSPAC G0 Partners: A cohort profile. Wellcome Open Res. 2023 Jan 24;8:37.

14. Bellou V, Belbasis L, Evangelou E. Tobacco Smoking and Risk for Pulmonary Fibrosis: A Prospective Cohort Study From the UK Biobank. Chest. 2021 Sep 1;160(3):983–93.

15. Garies S, Cummings M, Quan H, McBrien K, Drummond N, Manca D, et al. Methods to improve the quality of smoking records in a primary care EMR database: exploring multiple imputation and pattern-matching algorithms. BMC Med Inform Decis Mak. 2020 Mar 14;20(1):56.

16. Khouja JN, Taylor AE, Munafò MR. Associations between reasons for vaping and current vaping and smoking status: Evidence from a UK based cohort. Drug and Alcohol Dependence. 2020 Dec 1;217:108362.

17. Narkiewicz K, Kjeldsen SE, Hedner T. Is smoking a causative factor of hypertension? Blood Pressure. 2005 Apr 1;14(2):69–71.

18. Bowden J, Hemani G, Davey Smith G. Invited Commentary: Detecting Individual and Global Horizontal Pleiotropy in Mendelian Randomization—A Job for the Humble Heterogeneity Statistic? Am J Epidemiol. 2018 Dec 1;187(12):2681–5.

19. MendelianRandomization v0.9.0: updates to ... | Wellcome Open Research [Internet]. [cited 2024 Jul 23]. Available from: https://wellcomeopenresearch.org/articles/8-449#ref-20

20. Baum CF, Schaffer ME, Stillman S. Instrumental Variables and GMM: Estimation and Testing. The Stata Journal. 2003 Mar 1;3(1):1–31.

21. Yavorska OO, Burgess S. MendelianRandomization: an R package for performing Mendelian randomization analyses using summarized data. Int J Epidemiol. 2017 Dec 1;46(6):1734–9.

22. Zhao Q, Wang J, Hemani G, Bowden J, Small DS. Statistical inference in two-sample summary-data Mendelian randomization using robust adjusted profile score. The Annals of Statistics. 2020 Jun;48(3):1742–69.

23. Mitchell R, Hemani G, Dudding T, Corbin L, Harrison S, Paternoster L. UK Biobank Genetic Data: MRC-IEU Quality Control, version 2 [Internet]. [cited 2022 Jun 22]. Available from: https://research-information.bris.ac.uk/en/datasets/uk-biobank-genetic-data-mrc-ieu-quality-control-version-2
